# A Calcificação do Arco Aórtico Observada na Radiografia de Tórax Pode Servir Como um Preditor Independente de Acidente Vascular Cerebral Recorrente

**DOI:** 10.36660/abc.20230805

**Published:** 2024-07-10

**Authors:** Fahri Çakan, Asli Sert Sunal, Adem Adar, Orhan Onalan

**Affiliations:** 1 Çerkezköy State Hospital Tekirdağ Turquia Çerkezköy State Hospital, Tekirdağ – Turquia; 2 Baskent University Faculty of Medicine Alanya Application And Research Center Antalya Turquia Baskent University Faculty of Medicine – Alanya Application And Research Center, Antalya – Turquia; 3 Karabuk University Faculty of Medicine Karabuk Turquia Karabuk University Faculty of Medicine, Karabuk – Turquia

**Keywords:** Aorta Torácica, Acidente Vascular Cerebral, Eritrócitos

## Abstract

**Fundamento:**

Apesar dos avanços nas modalidades de diagnóstico e tratamento, há necessidade de marcadores preditivos para acidente vascular cerebral (AVC) recorrentes.

**Objetivos:**

Este estudo teve como objetivo investigar a relação entre calcificação do arco aórtico (CAA) e recorrência de AVC em pacientes com AVC durante o acompanhamento de um ano.

**Métodos:**

Todos os pacientes com AVC que sofreram seu primeiro evento foram avaliados para participação no estudo. Foram registrados pacientes que sofreram AVC recorrentes durante o acompanhamento de um ano. A CAA foi avaliada por radiografia de tórax. Com base na ocorrência de AVC recorrente, os pacientes foram divididos em dois grupos. A CAA foi classificada em quatro categorias de acordo com o seu grau, e a presença de CAA foi incluída na análise estatística. A relação entre CAA e AVC recorrente foi avaliada por meio de uma curva característica de operação do receptor. Um nível de significância <0,05 foi considerado aceitável para todas as análises estatísticas.

**Resultados:**

Um total de 203 pacientes foram incluídos no estudo (46,8% mulheres, média de idade 69±12,3). AVC recorrente foi detectado em 49 pacientes. CAA, hipertensão e fibrilação atrial foram mais frequentes em pacientes com AVC recorrente. Pacientes com AVC recorrente apresentaram menor taxa de filtração glomerular e maior largura de distribuição de glóbulos vermelhos (RDW). Na análise de regressão multivariada, CAA (hazard ratio [HR], 3,544; IC 95%:1,653-7,598, p=0,001) e RDW (HR,1,214; IC 95%:1,053-1,400, p=0,008) foram identificados como preditores independentes de AVC recorrente.

**Conclusão:**

A presença de CAA (≥ grau 1) e RDW foram significativamente associadas ao desenvolvimento de AVC recorrente dentro de um ano. Esses achados podem ter significado prognóstico no acompanhamento de pacientes com AVC.

## Graphical abstract

**Figura Central:**
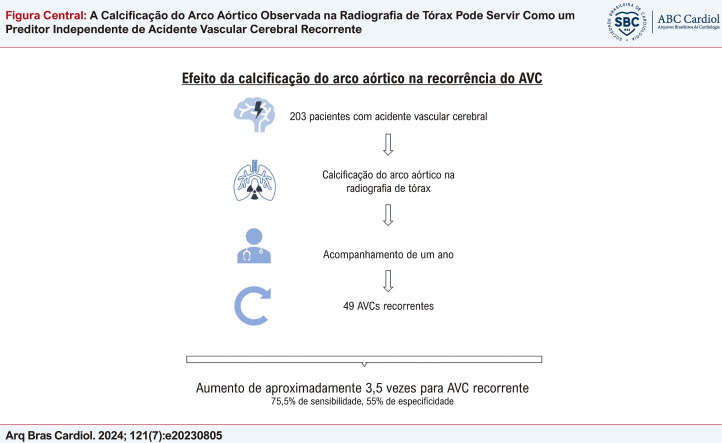
: A Calcificação do Arco Aórtico Observada na Radiografia de Tórax Pode Servir Como um Preditor Independente de Acidente Vascular Cerebral Recorrente

## Introdução

Apesar dos avanços no diagnóstico e tratamento, a carga das doenças cardiovasculares permanece elevada em todo o mundo.^1^ A prevenção de doenças cerebrovasculares tornou-se uma importante área de estudo na prática contemporânea. O acidente vascular cerebral (AVC) é um evento cerebrovascular comum com carga significativa de morbidade e mortalidade na população de pacientes. A etiologia do AVC isquêmico é atribuída a um evento trombótico ou embólico que leva à diminuição do fluxo sanguíneo para o cérebro. Seja trombótico ou embólico, a etiologia do AVC afeta tanto o prognóstico quanto os resultados. Nos últimos 50 anos, a incidência de AVC e as taxas de mortalidade pós-AVC diminuíram significativamente nos países de rendimento elevado, principalmente devido às mudanças nos fatores de risco cardiovascular e os avanços no tratamento do AVC agudo. Além disso, o AVC isquêmico recorrente tem sido associado ao aumento da mortalidade e à dependência funcional, embora esta área continue a ser insuficientemente investigada.^2^ Estudos mostraram taxas de recorrência variadas, desde 7-20% dentro de um ano para 16-35% dentro de cinco anos.^3^

A radiografia de tórax faz parte da rotina do exame cardiovascular e é um teste simples, facilmente acessível e comumente usado. Isso fornece informações significativas aos médicos sobre doenças do parênquima pulmonar e diversas condições cardiovasculares. Por exemplo, calcificação do arco aórtico (CAA) surge da inflamação e calcificação do arco aórtico resultante da progressão do dano endotelial e da hipertensão arterial.^4,5^ Descobriu-se que a CAA está associada a vários fatores de risco cardiovascular e tem significado clínico em eventos trombóticos. Exemplos incluem aterosclerose, síndrome coronariana aguda, AVC, eventos cardíacos adversos importantes e fibrilação atrial.^6-9^ É um parâmetro importante para avaliar riscos e possíveis complicações relacionadas à saúde cardiovascular.^10^ Foram identificadas relações estatisticamente significativas entre o grau de CAA, gravidade da doença cardiovascular e mortalidade. A extensão da CAA correlaciona-se com a gravidade da doença cardiovascular e tem implicações prognósticas para a mortalidade.^11^ A detecção de CAA nesses pacientes pode servir como fator orientador na identificação de potenciais eventos cerebrovasculares e na previsão de recorrência. Este estudo teve como objetivo investigar a relação entre a presença e o grau de CAA e o AVC recorrente.

## Métodos

### Design de estudo

Este estudo de coorte prospectivo foi realizado no Hospital Estadual Cerkezkoy do Ministério da Saúde entre janeiro de 2022 e junho de 2022. O consentimento informado por escrito foi obtido de todos os participantes seguindo os princípios éticos da pesquisa com seres humanos descritos na Declaração de Helsinque. Este estudo foi aprovado pelo Comitê de Ética em Pesquisa Clínica Não Intervencionista do Tekirdag City Hospital (ID #26). Todos os pacientes com idade≥ 18 anos que consentiram participar foram avaliados para inclusão neste estudo. Os critérios de exclusão foram os seguintes: história prévia de AVC, ataque isquêmico transitório, AVC hemorrágico, malignidade, gravidez, infecção ativa e achados radiográficos de tórax inadequados. Os pacientes foram acompanhados por um período de um ano por meio de consultas ambulatoriais. Os indivíduos que desenvolveram eventos cerebrovasculares recorrentes durante o período de acompanhamento também foram registrados. Os pacientes foram divididos ema dois grupos com base na incidência de acidentes cerebrovasculares e acidentes cerebrovasculares recorrentes.

### Parâmetros clínicos

Os fatores de risco cardiovascular de todos os pacientes foram examinados. A história de doença arterial coronariana, doença renal crônica, e AVC foi registrada. Pacientes que já haviam recebido antidiabéticos orais e/ou tratamento com insulina ou tiveram um nível de glicemia de jejum ≥126 mg/dL duas vezes foi considerado diabético. Foram considerados hipertensos pacientes que já haviam recebido tratamento anti-hipertensivo ou apresentavam pressão arterial ≥130/80 mmHg pelo menos duas vezes. Pacientes com nível de colesterol total >200 mg/dL, nível de colesterol de lipoproteína de baixa densidade (LDL) >100 mg/dL ou uso de hipolipemiantes foram considerados hiperlipidêmicos.^12^ A taxa de filtração glomerular estimada (TFG) foi calculada usando a equação da Chronic Kidney Disease Epidemiology Collaboration (CKD-EPI).^13^ O índice de massa corporal (IMC) foi calculado por meio dos valores do peso corporal (kg) dividido pela altura ao quadrado (m) (Índice de Quetelet). A área de superfície corporal (ASC) foi calculada como a raiz quadrada do produto do peso (kg) e altura (cm) dividido por 3.600.^14^ O uso de álcool, tabaco e medicamentos dos participantes foi registrado.

Testes bioquímicos de rotina, perfis lipídicos, testes de função tireoidiana e dados de hemograma completo foram registrados para todos os participantes. O estado do ritmo foi classificado como ritmo sinusal ou fibrilação/flutter atrial. Para os indivíduos com ritmo sinusal, foi realizada monitorização do ritmo-holter para detectar possíveis eventos arrítmicos e registrados aqueles com fibrilação atrial.

### Parâmetros ecocardiográficos

O exame ecocardiográfico transtorácico foi realizado em todos os pacientes utilizando um transdutor de 2,5-3,25 MHz (sistema Philips Affiniti 50 S4-2 Probe, Andover-EUA) seguindo as recomendações da Sociedade Americana de Ecocardiografia.^15-17^ A fração de ejeção do ventrículo esquerdo foi calculada usando o método de Simpson modificado.^18^ A massa ventricular esquerda (g) foi calculada pela fórmula de Devereux.^19^ O índice de massa ventricular esquerda foi calculado dividindo a massa ventricular esquerda pela área de superfície corporal. A hipertrofia ventricular esquerda foi definida como um índice de massa ventricular esquerda >95 g/m^2^ para mulheres e >115 g/m^2^ para homens. A espessura relativa da parede foi calculada como o dobro da espessura da parede posterior dividida pelo diâmetro diastólico do ventrículo esquerdo, e a geometria do ventrículo esquerdo foi categorizada em quatro categorias: geometria normal, remodelação concêntrica, hipertrofia concêntrica e hipertrofia excêntrica.^20^

### Avaliação de radiografia de tórax

**Figura 1:**
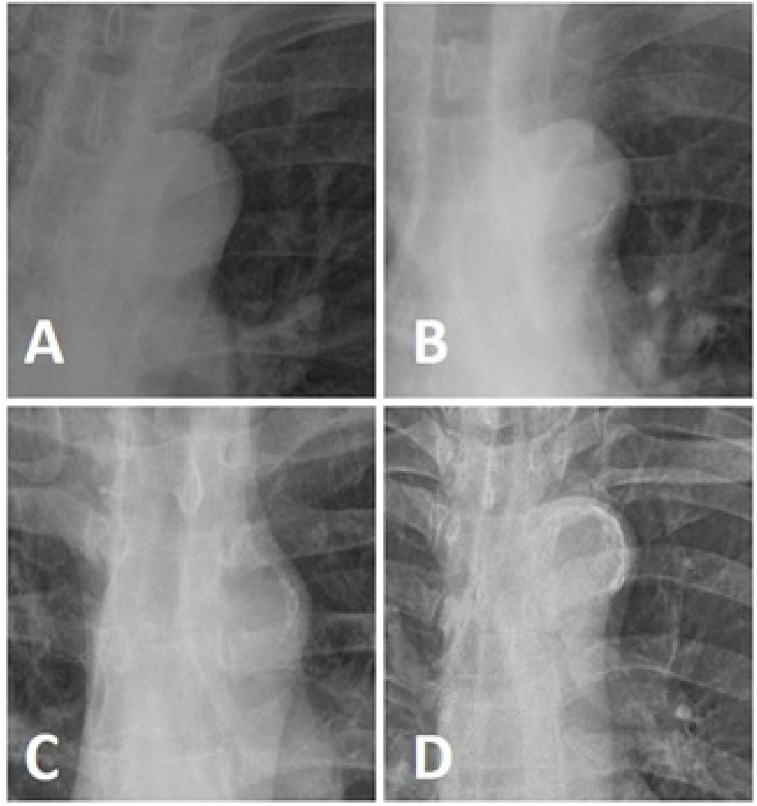
– Escore de quatro graus para calcificação do arco aórtico. A) Sem calcificação, B) Calcificação grau I, C) Calcificação grau II, D) Calcificação grau III.

Radiografia de tórax póstero-anterior foram obtidas enquanto o paciente estava em pé. A distância focal do paciente foi de 150 cm. Foi utilizado controle de exposição automatizado com tensão de tubo fixa de 117 kV.A CAA foi graduada da seguinte forma: grau 0, sem calcificação visível; grau 1, pequenas manchas de calcificação ou calcificação fina no arco aórtico; grau 2, uma ou mais áreas de calcificação espessada; e grau 3, calcificação circular do arco aórtico (Figura 1).^21^

### Parâmetros de traço

A classificação *Trial of Org 10172 in Acute Stroke Treatment (TOAST*) foi usada como quadro etiológico para AVC isquêmico, e os pacientes foram avaliados em cinco subgrupos:^22^ 1) aterosclerose de grandes artérias, 2) cardioembolismo, 3) oclusão de pequenos vasos, 4) AVC de outra etiologia determinada e 5) AVC de etiologia indeterminada. A gravidade do AVC foi determinada usando o *National Institutes of Health Stroke Score*.^23^ A classificação de Bamford^24^ foi utilizada para avaliar o território vascular afetado dividindo-o em quatro classes: AVC de circulação anterior total, AVC de circulação anterior parcial, síndrome lacunar e síndrome de circulação posterior. A escala de Rankin modificada (mRS) foi usada para avaliar a incapacidade pós-AVC e avaliar a recuperação funcional.^25^

### Análise estatística

O software IBM SPSS Statistics (IBM Corp. Lançado em 2011. IBM SPSS Statistics for Windows, Versão 20.0. Armonk, NY: IBM Corp) foi usado para todas as análises estatísticas. A distribuição normal das variáveis contínuas foi avaliada pelo exame visual dos histogramas, gráficos Q–Q e o teste de Kolmogorov-Smirnov. As variáveis contínuas com distribuição normal foram apresentadas como média (± desvio padrão), as variáveis contínuas com distribuição não normal como mediana (intervalo interquartil) e as variáveis categóricas como números e porcentagens. O teste t de Student (não pareado) foi utilizado para comparar variáveis contínuas com distribuição normal entre os dois grupos, enquanto o teste U de Mann-Whitney foi utilizado para variáveis contínuas com distribuição não normal. As variáveis categóricas foram comparadas pelos testes qui-quadrado ou exato de Fisher. O valor Kappa foi utilizado para calcular a variabilidade interobservador. A análise de regressão logística foi realizada para comparar a relação entre CAA e eventos cerebrovasculares recorrentes. Na análise de regressão univariada, um valor de p bilateral inferior a 0,1 foi considerado estatisticamente significativo para inclusão na análise de regressão multivariada. Os testes de Pearson ou Spearman foram usados para analisar as correlações entre os parâmetros. A relação entre CAA e eventos cerebrovasculares recorrentes foi avaliada por meio de uma análise da curva *Receiver Operating Characteristic* (ROC). Um valor p bilateral <0,05 foi considerado estatisticamente significativo para todas as comparações.

## Resultados

**Figura 2:**
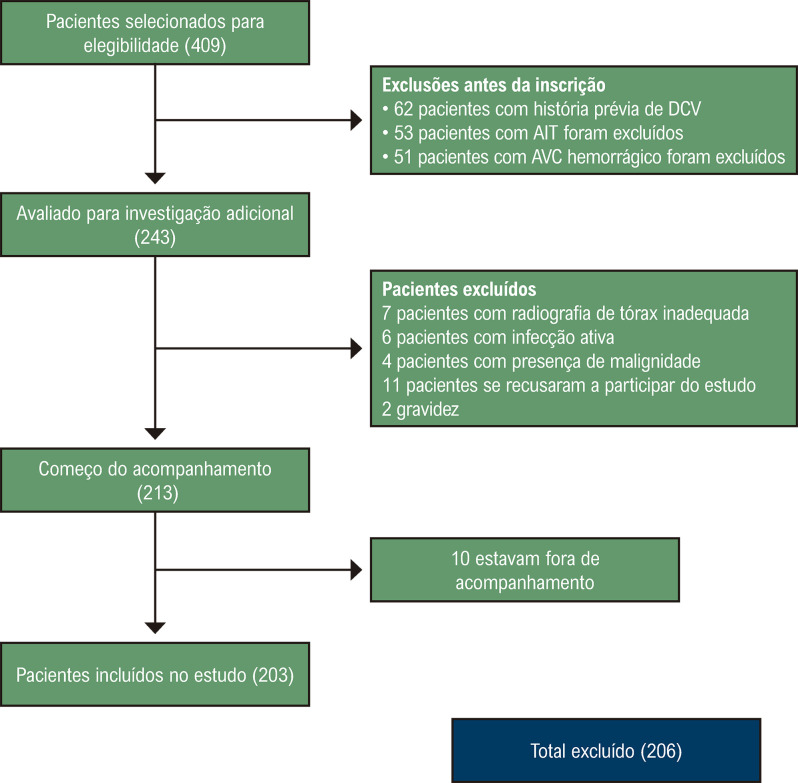
– Fluxograma de seleção de pacientes. DCV: doença cardiovascular; AIT: ataque isquêmico transitório; AVC: acidente vascular cerebral.

No total, 409 pacientes foram incluídos no estudo durante o período especificado. Os seguintes pacientes foram excluídos do estudo: história anterior de AVC (n=62), ataque isquêmico transitório (n=53), AVC hemorrágico (n=51), malignidade (n=4), gravidez (n=2), infecção ativa (n=6), e radiografia de tórax inadequada (n=7). Onze pacientes se recusaram a participar do estudo e 10 foram perdidos no acompanhamento. Os 203 pacientes restantes foram incluídos no estudo (Figura 2). Destes, 95 eram do sexo feminino, representando 46,8% do total da população. A idade média dos participantes foi de 69 (±12,3) anos. As características demográficas básicas dos participantes do estudo são apresentadas na Tabela 1.

**Tabela 1:** – Características clínicas e demográficas da amostra

Característica	Total (n=203)	AVC recorrente (n=154)	AVC não recorrente (n=49)	p
Gênero (feminino), n (%)	95 (46,8%)	72 (46,8%)	23 (46,9%)	0,556
Idade (ano)	69 (±12,3)	68,4 (±12,6)	71,1 (±11,2)	0,231
Peso (kg)	75 (15)	76 (15)	75 (14)	0,964
Altura (cm)	165 (12)	166 (12)	165 (16)	0,935
IMC (kg/m^2^)	27,1 (5,1)	27,0 (5,5)	27,1 (4,0)	0,958
ASC (m^2^)	1,87 (±0,17)	1,87 (±0,17)	1,86 (±0,17)	0,941
Hipertensão, n (%)	156 (77,2%%)	110 (71,4%)	46 (93,9%)	0,001
Diabetes Mellitus, n (%)	75 (36,9%)	53 (34,4%)	22 (44,9%)	0,125
Doença Arterial Coronariana, n (%)	42 (20,7%)	30 (19,5%)	12 (24,5%)	0,286
Doença Renal Crônica, n (%)	10 (4,9%)	7 (4,5%)	3 (6,1%)	0,45
Hiperlipidemia, n (%)	97 (47,8%)	73 (47,4%)	24 (49%)	0,488
Cigarro, n (%)	57 (28,1%)	41 (26,6%)	16 (32,7%)	0,26
Álcool, n (%)	19 (9,4%)	15 (9,7%)	4 (8,2%)	0,497
Inibidor do Sistema Renina-Angiotensina, n (%)	122 (60,1%)	84 (54,5%)	38 (77,6%)	0,003
Estatina, n (%)	119 58,6%)	91 (59,1%)	28 (57,1%)	0,468
Bloqueador de canais de cálcio, n (%)	61 (30%)	42 (27,3%)	19 (38,8%)	0,09
Betabloqueador, n (%)	76 (37,4%)	54 (35,1%)	22 (44,9%)	0,143
Insulina, n (%)	17 (8,4%)	12 (7,8%)	5 (10,2%)	0,392
Anticoagulante, n (%)	34 (16,7%)	20 (13%)	14 (28,6%)	0,012
Fibrato, n (%)	3 (1,4%)	1 (0,6%)	2 (4,1%)	0,145
Antiplaquetário, n (%)	78 (38,8%)	58 (37,7%)	20 (40,8%)	0,433
Ácido Acetilsalicílico, n (%)	61 (30%)	108 (70,1%)	34 (69,4%)	0,527
Antidiabético Oral, n (%)	62 (30,5%)	43 (27,9%)	19 (38,8%)	0,105
Fibrilação Atrial, n (%)	42 (20,7%)	34 (22,1%)	15 (30,6%)	0,042
Escore CHA_2_DS_2_-VASc	4 (1)	4 (2)	5 (2)	<0,001
Calcificação do Arco Aórtico				
0	98 (48,3%)	86 (55,8%)	12 (24,5%)	<0,001
1	71 (35%)	49 (31,8%)	22 (44,9%)	
2	31 (15,3%)	17 (11%)	14 (28,6%)	
3	3 (1,5%)	2 (1,3%)	1 (2,0%)	
≥1	105 (51,7%)	68 (44,1%)	37 (75,5%)	<0,001
≥2	24 (16,7%)	19 (12,3%)	15 (30,6%)	0,004
Classificação de Bamford				
LACS, n (%)	59 (29,1%)	49(31,2%)	10(20,4%)	
PACS, n (%)	83 (40,9%)	55(35,7%)	28(57,1%)	
POCS, n (%)	46 (22,7%)	38(24,7%)	8(16,3%)	0,118
TACS, n (%)	14 (6,9%)	11(7,1%)	3(6,1%)	
Classificação TOAST				
Aterosclerose de grandes artérias, n (%)	54 (26,6%)	37 (24,0%)	17 (34,7%)	
Lacunar, n (%)	42 (20,7%)	28 (18,2%)	14 (28,6%)	
Cardioembolismo, n (%)	20 (9,9%)	17 (11,0%)	3 (6,1%)	
Outro, n (%)	5 (2,5%)	3 (1,9%)	2 (4,0%)	0,138
Indeterminado, n (%)	81 (39,9%)	68 (44,1%)	13 (26,5%)	
Escala de Rankin Modificada	2 (3)	1 (3)	2 (2)	0,021
Escala de AVC do NIH	3 (5)	3 (4)	5 (7)	0,047

*IMC: índice de massa corporal; ASC: área de superfície corporal; AVC: acidente vascular cerebral.*

Neste estudo, 156 indivíduos (77,2%) apresentavam hipertensão, e essa proporção foi significativamente maior no grupo de evento cerebrovascular recorrente (93,9%, 46 indivíduos) do que no grupo não recorrente (110 indivíduos, 71,4%) (p<0,001). Além disso, o uso de bloqueadores do sistema renina-angiotensina (77,6%) e anticoagulantes (28,6%) foi significativamente maior no grupo de eventos recorrentes do que nos demais grupos (p=0,003 e p=0,012, respectivamente). A fibrilação atrial foi detectada em 34 indivíduos (22,1%) no grupo de primeiro evento cerebrovascular e em 15 indivíduos (30,6%) no grupo de evento recorrente, mostrando diferença significativa (p=0,042). Além disso, o escore CHA_2_DS_2_-VASc calculado, independentemente do ritmo, foi maior no grupo de eventos cerebrovasculares recorrentes, com mediana de 5 (2), do que nos demais grupos (p<0,001).

Ao final do primeiro ano de acompanhamento, foi observado AVC recorrente em 49 pacientes (24,1%) pacientes. Os pacientes foram divididos em dois grupos com base no status de recorrência do AVC (AVC não recorrente, 154 pacientes; AVC recorrente, 49 pacientes). Os dois grupos tinham características demográficas semelhantes. Uma comparação dos dois grupos é apresentada na Tabela 1. Em relação às características neurológicas, o AVC parcial de circulação anterior foi o subtipo mais comum de AVC. Foi encontrado em 55 indivíduos (35,7%) no grupo de AVC não recorrente e em 28 indivíduos (57,1%) no grupo de evento recorrente. Os grupos foram semelhantes segundo a classificação de Bamford. Pela classificação TOAST, predominaram casos de causa desconhecida em todo o grupo, o que foi notável. Esse padrão foi observado não apenas em indivíduos que vivenciaram seu primeiro evento cerebrovascular mas também em 17 indivíduos (34,7%) com aterosclerose de grandes artérias e 14 indivíduos (28,6%) com infartos lacunares entre aqueles com eventos cerebrovasculares recorrentes. Os casos de AVC com etiologia indeterminada ficaram em terceiro lugar. No entanto, essas diferenças não eram estatisticamente muito significativas. Os escores Rankin modificado e o da NIH Stroke Scale foram maiores no grupo de eventos cerebrovasculares recorrentes (p=0,021 e 0,047, respectivamente).

A CAA foi comparada entre os grupos. A CAA foi detectada em 105(51,7%) pacientes. Houve uma diferença significativa nos graus da CAA entre os grupos (p<0,001). Um paciente (2%) apresentou CAA grau 3, e 14 (28,6%) pacientes apresentaram CAA grau 2 no grupo AVC recorrente. Esses números foram de apenas 2 (1,3%) com CAA grau 3, e 17 (11%) com CAA grau 2 no grupo de evento cerebrovascular não recorrente. Portanto, os pacientes com CAA foram combinados e analisados como um único grupo. AAC (grau ≥1) foi observada em 37 indivíduos (75,5%) no grupo de eventos cerebrovasculares de AAC e em 68 (44,1%) no grupo de eventos cerebrovasculares não recorrentes. O grupo de eventos cerebrovasculares recorrentes apresentou prevalência de CAA significativamente maior (p<0,001). Entre os participantes, 60 radiografias de tórax selecionadas aleatoriamente foram avaliadas por dois cardiologistas e um neurologista que desconheciam os achados do estudo quanto à variabilidade interobservador e houve um grau razoavelmente alto de consistência entre as avaliações. (Valor Kappa = 0,816, p < 0,001).

As características laboratoriais e ecocardiográficas de cada grupo são mostrados na Tabela 2. No grupo de eventos cerebrovasculares recorrentes, a taxa de filtração glomerular (TFG) foi estatisticamente significativamente menor em comparação ao grupo de eventos cerebrovasculares não recorrentes [77 mL/min/1,73 m^2^ (33) vs. /1,73 m2 (23), p=0,018]. Além disso, a largura de distribuição de glóbulos vermelhos (RDW) foi significativamente maior no grupo de eventos cerebrovasculares recorrentes (16,3 [3,8] fL) do que no grupo de eventos cerebrovasculares não recorrentes (15,2 [2,1] fL) (p=0,001). Não foram observadas diferenças estatisticamente significativas entre os grupos em relação aos demais parâmetros laboratoriais.

**Tabela 2:** – Achados laboratoriais e ecocardiográficos da população estudada

Parâmetros	AVC não recorrente	AVC recorrente	p
**Parâmetros laboratoriais**			
Glicose (mg/dL)	111 (50)	117 (40)	0,541
TFG (mL/min/1,73 m^2^)	85 (23)	77 (33)	0,018
Alanina Aminotransferase (U/L)	15 (11)	15 (18)	0,737
Aspartato Aminotransferase (U/L)	18 (8)	18 (13)	0,372
Sódio (mEq/L)	140 (3)	140 (5)	0,657
Potássio (mEq/L)	4,3 (±0,5)	4,3 (±0,5)	0,768
Triglicerídeos (mg/dL)	135 (98)	128 (74)	0,916
Colesterol total (mg/dL)	186 (±44,4)	178 (±51)	0,185
Lipoproteína de baixa densidade (mg/dL)	111 (±37,3)	108 (±41,4)	0,561
Lipoproteína de alta densidade (mg/dL)	42 (18)	42 (16)	0,632
Glóbulo Branco (n, x103)	8,15 (±2,44)	8,20 (±2,87)	0,924
Hemoglobina (g/dL)	13,0 (±2)	12,5 (±2,3)	0,112
Plaquetas (n, x103)	233 (96)	249 (84)	0,052
PDW (%)	16,7 (±2,6)	16,5 (±2,5)	0,509
Crítico de plaquetas (%)	0,22 (0,07)	0,24 (0,09)	0,047
Neutrófilo (n, x103)	4,75 (2,22)	4,60 (2,88)	0,881
Linfócito (n, x103)	2,05 (±0,69)	1,95 (±0,86)	0,141
RDW (fL)	15,2 (2,1)	16,3 (3,8)	0,001
T3 livre (ng/L)	2,45 (0,64)	2,37 (0,80)	0,261
T4 livre (ng/dL)	1,02 (0,20)	1,03 (0,22)	0,528
TSH (mUI/L)	1,36 (1,32)	1,33 (1,28)	0,885
**Parâmetros ecocardiográficos**			
Diâmetro diastólico final do ventrículo esquerdo (mm)	44 (4)	44 (4)	0,4
Diâmetro Sistólico Final do Ventrículo Esquerdo (mm)	29 (4)	29 (4)	0,691
Fração de Ejeção Ventricular Esquerda (%)	59,88 (6,3)	59,6 (8,2)	0,75
Massa Ventricular Esquerda (gr)	203,05 (69)	207,28 (67)	0,949
Índice de Massa Ventricular Esquerda (gr/m^2^)	109,49 (31,7)	109 (38,1)	0,991
Espessura relativa da parede	0,54 (±0,07)	0,55 (±0,07)	0,574
Diâmetro Aórtico (mm)	33,46 (±3,9)	33,49 (±4,3)	0,908
Espessura do Septo Interventricular (mm)	12 (2)	13 (3)	0,36
Espessura da Parede Posterior (mm)	12 (2)	12 (2)	0,563
Diâmetro Atrial Esquerdo (mm)	36 (5)	37 (4)	0,275
Pressão estimada da artéria pulmonar (mmHg)	9 (15,8)	16 (17,8)	0,009
Regurgitação aórtica (≥moderado), n (%)	2 (1,3)	3 (6,1)	0,058
Insuficiência mitral (≥moderado), n (%)	4 (2,6)	3 (6,1)	0,363
Regurgitação Tricúspide (≥moderado), n (%)	6 (3,9)	2 (4,1)	0,616
**Geometria Ventricular Esquerda**			
Hipertrofia Concêntrica	82 (53,2)	26 (53,1)	
Remodelação Concêntrica	64 (41,6)	21 (42,9)	0,991
Hipertrofia Excêntrica	4 (2,6)	1 (2)	
Geometria Normal	4 (2,6)	1 (2)	

*TFG: taxa de filtração glomerular; RDW: Glóbulos Vermelhos; PDW: Largura de distribuição plaquetária; TSH: hormônio estimulador da tireoide.*

Os grupos foram semelhantes em termos de parâmetros ecocardiográficos, exceto pela pressão estimada da artéria pulmonar. A pressão estimada da artéria pulmonar foi maior no grupo com AVC recorrente [16 (±17,8) mmHg] do que no grupo com AVC não recorrente [9 (±15,8) mmHg]), e essa diferença foi estatisticamente significativa (p=0,009).

CAA, RDW e regurgitação aórtica foram associadas à recorrência de AVC na análise de regressão logística univariada (p<0,1) (Tabela 3). Na análise de regressão multivariada, foi encontrada associação independente e forte entre CAA (OR 3,544, p<0,001), RDW (OR 1,214, p=0,008) e AVC (Tabela 3, Figura Central).

**Tabela 3:** – Análise univariada e multivariada para padrão pressórico não-dipper

Parâmetro	Análise univariada		Análise multivariada		
	β	p	β	p	Taxa de risco (IC 95%)
Hipertensão	1.239	0,130			
Fibrilação atrial	-0,286	0,751			
Calcificação do Arco Aórtico (≥1)	1.260	0,007	1.265	0,001	3,544 (1,653 - 7,598)
Taxa de filtração glomerular	-0,008	0,432			
Pressão estimada da artéria pulmonar	0,015	0,190			
Escala de AVC do NIH	-0,041	0,567			
Escala de Rankin Modificada	0,201	0,398			
Anticoagulante	0,863	0,174			
Inibidor RAS	0,259	0,621			
Escore CHA_2_DS_2_-VASc	-0,128	0,564			
RDW	0,135	0,070	0,194	0,008	1,214 (1,053-1,400)
Regurgitação aórtica	2.270	0,097	2.376	0,066	10,766 (0,857 - 135,171)
Constante	-4.652	0,004			

*IC: Intervalo de Confiança; AVC: acidente vascular cerebral; RDW: glóbulos vermelhos.*

A análise de correlação foi realizada para investigar melhor a associação potencial entre CAA e RDW. Pacientes com CAA tiveram maior RDW (p=0,014). Não houve correlação entre o grau da CAA e o RDW (p=0,055, r=0,135). No entanto, houve correlação moderada entre o grau de CAA e AVC recorrente (p<0,001, r=0,277). Estes resultados sugerem que a RDW não é um fator de confusão significativo.

**Figura 3:**
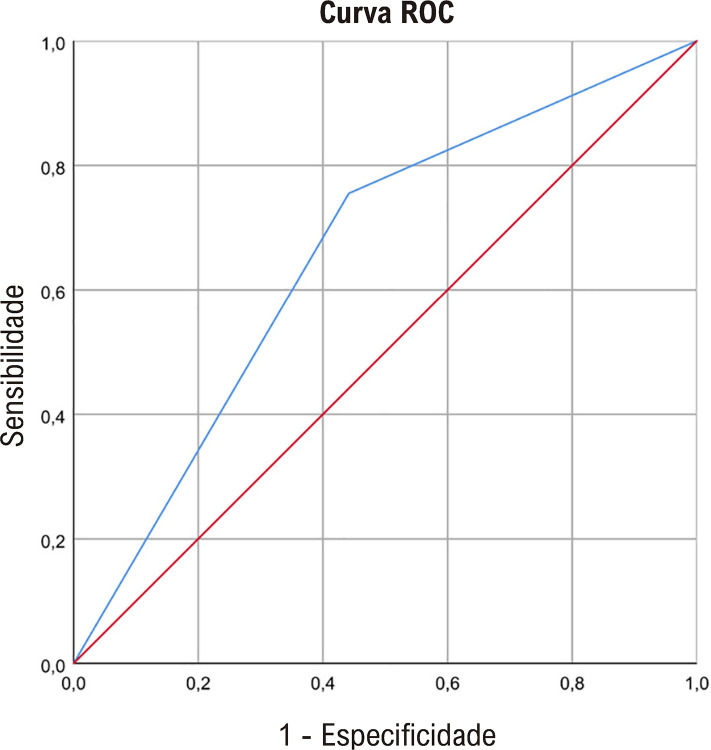
– Curva característica de operação do receptor de calcificação do arco aórtico para acidente vascular cerebral recorrente.

A análise da curva ROC produziu uma forte capacidade preditiva de CAA grau ≥ 1 para AVC recorrente (AUC=0,657, p<0,001) (Figura 3). A presença de CAA na radiografia de tórax apresentou sensibilidade e especificidade de 75,5% e 55,8%, respectivamente, para AVC recorrente (Tabela 4).

**Tabela 4:** – Área sob a curva para calcificação do arco aórtico

AUC	p	IC 95%	Sensibilidade	Especificidade	VPP	VPN
0,657	0,001	0,572 - 0,742	0,755	0,558	33,33%	87,76%

*AUC: área sob a Curva; IC: intervalo de confiança; VPP: valor preditivo positivo; VPN: valor preditivo negativo.*

## Discussão

Este estudo teve como objetivo avaliar potenciais fatores de risco para AVC recorrente e determinar sua associação com a CAA. Consistente com esses dados, a calcificação aórtica, que é um subgrupo específico desta condição, foi considerada um preditor de ambos incidentes e AVC recorrente, que é um subgrupo específico desta condição.^7^ Além disso, foi demonstrado que o valor do RDW é maior em pacientes com AVC recorrente. Essas descobertas podem servir como diretrizes clínicas.

Este estudo incluiu pacientes com qualquer forma de AVC. Portanto, espera-se que não haja diferença significativa em fatores de risco clássicos. No entanto, uma descoberta interessante é digna de nota nos resultados. No grupo de AVC recorrente, a hipertensão foi significativamente mais prevalente em um nível estatisticamente significativo. A hipertensão foi o fator de risco cardiovascular mais comum. Embora seja geralmente considerado o fator de risco mais importante para o primeiro AVC, o seu papel no risco de recorrência permanece obscuro. Embora seja um fator de risco independente para eventos cerebrovasculares, também representa um risco dentro do espectro de eventos cerebrovasculares. Se o subagrupamento e a classificação fossem realizados para outros fatores de risco, diferenças poderiam ter sido detectadas. No entanto, é importante ter em mente que a hipertensão continua a ser um fator de risco para acidentes vasculares cerebrais. Acreditamos que a diferença observada no uso de bloqueadores do SRA entre os grupos também decorre desta entidade clínica.

A maior incidência de fibrilação atrial e o maior uso de anticoagulantes no grupo de AVC recorrente foram considerados resultado da relação entre essas condições clínicas. Foi avaliado o escore CHA_2_DS_2_-VASc, independentemente da presença de FA nos grupos. Um escore mediano significativamente maior de CHA_2_DS_2_ – VASc foi observado no grupo de AVC recorrente. Conforme mostra a Tabela 1, além da hipertensão, os grupos eram semelhantes, e acreditamos que essa diferença pode ser atribuída à hipertensão, que é um componente do cálculo do escore CHA_2_DS_2_-VASc.

No grupo de AVC recorrente, foi observada menor TFG. Isso pode ser atribuído ao fato de os indivíduos do grupo de AVC recorrente apresentarem pior desempenho e maior morbidade, o que pode ter levado a uma maior prevalência de problemas nutricionais. Consequentemente, a perda de massa muscular pode afetar os níveis séricos de creatinina e a TFG.

Em uma grande metanálise que incluiu dez estudos, foram examinados preditores de AVC recorrente, e um histórico de AVC ou ataque isquêmico transitório (AIT) e a presença de aterosclerose significativa de grandes artérias foram associados a AVC recorrente. Nesta metanálise, os achados foram avaliados com base nos achados da ressonância magnética, e também foi discutida a ausência de um estudo que os avaliasse por meio de TC ou USG.^26^ Além desta metanálise, um estudo recente investigou a relação entre calcificação aórtica na tomografia computadorizada de tórax e eventos cerebrovasculares. Neste estudo, o grau CAA ≥ 1 foi associado a AVC recorrente.^27^ Este estudo também mencionou estudos realizados com raios X; no entanto, esses estudos não classificaram CAA da mesma maneira que no estudo atual. Além disso, condições especializadas como o escore de Agatston não foram incluídas neste estudo. Embora a tomografia computadorizada de tórax seja certamente superior à radiografia de tórax em corte transversal, considerando os achados obtidos em nosso estudo, pode-se argumentar que a radiografia de tórax é mais custo-efetiva. Mais pesquisas poderiam explorar a concordância entre estudos que avaliam as duas modalidades. Na verdade, as radiografias de tórax prontamente disponíveis podem ser usadas para determinar o risco de um paciente sofrer AVC recorrente. As radiografias de tórax costumam fazer parte das internações hospitalares de rotina em muitos ambientes clínicos.

Se considerarmos o conceito de “aterosclerose de grandes artérias” mencionado na metanálise, a aorta é o ponto de partida dessas artérias. A calcificação do arco aórtico é um importante indicador de aterosclerose de grandes artérias. A hipótese do nosso estudo é consistente com os achados desta meta-análise. Em nosso estudo, uma etiologia indeterminada foi mais comum no grupo de AVC não recorrente (44,1%), enquanto a aterosclerose de grandes artérias foi observada em maior taxa no grupo de AVC recorrente (34,7%). Essa diferença não foi estatisticamente significativa, possivelmente devido ao pequeno número de potenciais participantes (p=0,138). Outro ponto importante é que as radiografias de tórax são mais econômicas, envolvem menos exposição à radiação e são mais facilmente aplicáveis do que as modalidades tomográficas e angiográficas. Além disso, à luz da pandemia, as radiografias de tórax são realizadas rotineiramente em muitos centros de saúde durante a hospitalização como prática clínica preferencial.

A hipertensão tem um efeito significativo sobre o leito vascular. Primeiro, o aumento da pressão vascular leva à calcificação vascular, um importante indicador de dano vascular relacionado à hipertensão. O efeito oclusivo da hipertensão desempenha um papel na etiologia do AVC isquêmico. Adicionalmente, a carga de pressão pode causar ruptura e hemorragia de vasos cerebrais, bem como fornecimento de sangue prejudicado ao tecido cerebral devido a efeitos de compressão.^28^ Outros fatores que desempenham um papel na doença vascular oclusiva através da calcificação vascular incluem inflamação, estresse oxidativo, idade avançada e o sistema renina-angiotensina.^29-31^ As células musculares lisas vasculares, semelhantes aos osteoblastos, são derivadas de células mesenquimais. Sob a influência desses fatores, as células musculares passam por uma transição fenotípica para um fenótipo semelhante ao dos osteoblastos e produzem cálcio. Assim, a calcificação vascular começa com a produção de cálcio na íntima ou média das paredes dos vasos sanguíneos.^32^ A CAA leva ao aumento da rigidez arterial, resultando em diminuição da complacência vascular, e está associada à hipertrofia ventricular esquerda e disfunção diastólica.^33,34^ Como resultado, o fluxo sanguíneo cerebral diminui, levando a uma nutrição prejudicada nas regiões cerebrais relacionadas. Por exemplo, foi relatada uma forte correlação entre CAA e calcificação da artéria renal, um indicador significativo de doença arterial renal. Existem duas descobertas importantes sobre a relação entre AVC recorrente e CAA. CAA é um protótipo significativo de calcificação vascular, que é uma condição sistêmica que afeta todo o leito vascular. Portanto, é um indicador de risco em pacientes com AVC isquêmico. Isso indicou que o processo de calcificação era progressivo. Na verdade, esses pacientes sofreram AVC recorrentes. Este achado é importante para demonstrar danos em órgãos-alvo, que é um desfecho crucial em doenças cardiovasculares, pois resultou em desfechos clínicos, como AVC recorrente.

A largura de distribuição dos RDW reflete a heterogeneidade do volume dos glóbulos vermelhos. Em nosso estudo, foram encontrados valores mais elevados de RDW no grupo de AVC recorrente, e essa diferença foi estatisticamente significativa na previsão de AVC recorrentes. A relação entre RDW e o risco de AVC isquêmico, doença da artéria carótida e êmbolos cerebrais foi relatada anteriormente.^35,36^ Além disso, foi demonstrado que cada aumento de 1% no RDW está associado a um aumento de 13% no risco de AVC isquêmico.^37^ Nossos dados, consistentes com os de outros estudos, fornecem informações adicionais. Nossos achados são consistentes com os de um estudo recente de Shen et al., que utilizou dados de acompanhamento de 5 anos. Semelhante ao nosso estudo, Shen et al. demonstraram uma associação positiva entre altos níveis de RDW e aumento do risco de AVC isquêmico recorrente.^38^

A relação fisiopatológica exata entre RDW e AVC recorrente não foi estudada extensivamente. No entanto, semelhante à CAA, foi sugerido que a inflamação e o estresse oxidativo podem desempenhar um papel no mecanismo. Consequentemente, a taxa de sobrevivência dos glóbulos vermelhos diminui, e a produção de eritropoetina é inibida, resultando em aumento de níveis de RDW.^39^

Este estudo teve certas limitações. Primeiro, foi realizado em um único centro com uma amostra relativamente pequena, considerando as doenças cardiovasculares. Essas descobertas foram baseadas em dados observacionais e não puderam ser controladas para variáveis de confusão. Além disso, a relação causal entre CAA e AVC recorrente deve ser determinada de acordo com mecanismos fisiopatológicos. A CAA foi avaliada para sua presença; no entanto, outras características, como espessura total ou características de alto risco, como ulceração, não foram avaliadas. Além disso, os escores gerais do NIHSS foram de baixo a moderado, o que pode limitar a generalização dos resultados para pacientes com AVC mais graves. Por fim, foram excluídos pacientes com história de AVC e AIT, e esses dados podem não aplicar para esta população de pacientes.

## Conclusão

Neste estudo, foi observada uma relação estatisticamente significativa entre AVC recorrentes e CAA durante acompanhamento de um ano apesar do pequeno número de pacientes. Além disso, foi observada uma relação entre AVC recorrente e RDW. Ambos os parâmetros são facilmente acessíveis e clinicamente convenientes e podem trazer benefícios aos médicos no monitoramento de pacientes com AVC.

### Destaques

- O AVC recorrente é uma importante causa de morbidade e mortalidade.
- A calcificação do arco aórtico é um importante marcador clínico da carga de doença vascular.
- A calcificação do arco aórtico na radiografia de tórax pode ser um fator chave em acidentes vasculares cerebrais recorrentes.
- A RDW demonstrou ser um indicador eficaz de AVC recorrente.

### Ética

Este estudo atendeu os padrões internacionalmente aceitos para práticas de pesquisa e relatórios. Este estudo foi aprovado pelo Comitê de Ética em Pesquisa Clínica Não Intervencionista do Tekirdag City Hospital (ID #26). O consentimento informado por escrito foi obtido de todos os participantes seguindo os princípios éticos da pesquisa em seres humanos descritos na Declaração de Helsinque.
