# Supplementary material for: A Calcificação do Arco Aórtico Observada na Radiografia de Tórax Pode Servir Como um Preditor Independente de Acidente Vascular Cerebral Recorrente
Source: Arq Bras Cardiol. 2024 Jul 10;121(7):e20230805. [Article in Portuguese] doi: 10.36660/abc.20230805 (PMC12080711; doi:10.36660/abc.20230805)
Supplement: Translation

# Translation: Aortic Arch Calcification Observed on Chest X-Ray May Serve as an Independent Predictor for Recurrent Stroke

**DOI:** 10.36660/abc.20230805i

**Authors:** Fahri Çakan, Asli Sert Sunal, Adem Adar, Orhan Onalan

**Affiliations:** 1 Turkey Çerkezköy State Hospital, Tekirdağ – Turkey; 2 Turkey Baskent University Faculty of Medicine - Alanya Application And Research Center, Antalya – Turkey; 3 Turkey Karabuk University Faculty of Medicine, Karabuk – Turkey

**Keywords:** Thoracic Aorta, Stroke, Erythrocytes

## Abstract

**Background:**

Despite advances in diagnostic and treatment modalities, there is a need for predictive markers for recurrent strokes.

**Objectives:**

This study aimed to investigate the relationship between aortic arch calcification (AAC) and stroke recurrence in stroke patients during a one-year follow-up.

**Methods:**

All stroke patients who experienced their first event were evaluated for participation in the study. Patients who experienced recurrent strokes during the one-year follow-up were recorded. AAC was assessed by chest radiography. Based on the occurrence of recurrent strokes the patients were divided into two groups. AAC was classified into four categories according to its degree, and the presence of AAC was included in the statistical analysis. The relationship between AAC and recurrent stroke was assessed using a receiver operating characteristic curve. A significance level of <0.05 was deemed acceptable for all statistical analyses.

**Results:**

A total of 203 patients were included in the study (46.8% female, mean age 69±12.3). Recurrent stroke was detected in 49 patients. AAC, hypertension, and atrial fibrillation were more frequent in patients with recurrent stroke. Patients with recurrent stroke had a lower glomerular filtration rate and a higher red cell distribution width (RDW). In multivariate regression analysis, AAC (hazard ratio [HR], 3.544; 95% CI:1.653-7.598, p=0.001) and RDW (HR,1.214; 95% CI:1.053-1.400, p=0.008) were identified as independent predictors of recurrent stroke.

**Conclusion:**

The presence of AAC (≥ grade 1) and RDW were found to be significantly associated with the development of recurrent stroke within one year. These findings may have prognostic significance in the follow-up of stroke patients.

## Introduction

Despite advances in diagnosis and management, the burden of cardiovascular disease remains high worldwide.^1^ The prevention of cerebrovascular diseases has become an important area of study in contemporary practice. Stroke is a common cerebrovascular event with significant morbidity and mortality burden in the patient population. The etiology of ischemic stroke is attributed to a thrombotic or embolic event that leads to a decrease in blood flow to the brain. Whether thrombotic or embolic, the etiology of stroke affects both prognosis and outcomes. Over the past 50 years, the incidence of stroke and post-stroke mortality rates have significantly decreased in high-income countries, primarily because of the changes in cardiovascular risk factors and advancements in acute stroke treatment. Furthermore, recurrent ischemic stroke has been associated with increased mortality and functional dependency, although this area remains insufficiently researched.^2^ Studies have shown varying recurrence rates ranging from 7-20% within one year to 16-35% within five years.^3^

Chest radiography is a routine part of cardiovascular examination and is a simple, readily accessible, and commonly used test. This provides significant information to clinicians regarding lung parenchymal disease and various cardiovascular conditions. For instance, Aortic arch calcification (AAC) arises from inflammation and calcification of the aortic arch resulting from the progression of endothelial damage and high blood pressure.^4,5^ AAC has been found to be associated with numerous cardiovascular risk factors and holds clinical significance in thrombotic events. Examples include atherosclerosis, acute coronary syndrome, stroke, major adverse cardiac events, and atrial fibrillation.^6-9^ It is an important parameter for assessing risks and potential complications related to cardiovascular health.^10^ Statistically significant relationships were identified between the AAC degree, cardiovascular disease severity, and mortality. The extent of AAC correlates with cardiovascular disease severity and has prognostic implications for mortality.^11^ The detection of AAC in these patients can serve as a guiding factor in identifying potential cerebrovascular events and predicting recurrence. This study aimed to investigate the relationship between the presence and degree of AAC and recurrent stroke.

## Methods

### Study design

This prospective cohort study was performed at the Health Ministery Cerkezkoy State Hospital between January 2022 and June 2022. Written informed consent was obtained from all the participants following the ethical principles of human research outlined in the Declaration of Helsinki. This study was approved by the Tekirdag City Hospital Non-Interventional Clinical Research Ethics Committee (ID #26). All patients aged ≥ 18 years who consented to participate were evaluated for inclusion in this study. Exclusion criteria were as follows: previous stroke history, transient ischemic attack, hemorrhagic stroke, malignancy, pregnancy, active infection, and improper chest radiographic findings. Patients were followed up for a period of one year through outpatient clinic visits. Individuals who developed recurrent cerebrovascular events during the follow-up period were also recorded. Patients were divided into two groups based on the incidence of cerebrovascular accidents and recurrent cerebrovascular accidents.

### Clinical parameters

The cardiovascular risk factors of all patients were examined. A history of coronary artery disease, chronic kidney disease, and stroke was recorded. Patients who had previously received oral antidiabetic and/or insulin treatment or had a fasting blood glucose level of ≥126 mg/dL twice were considered diabetic. Patients who had previously received antihypertensive treatment or had a blood pressure of ≥130/80 mmHg at least twice were considered hypertensive. Patients with a total cholesterol level >200 mg/dL, low-density lipoprotein cholesterol (LDL) level >100 mg/dL, or the use of lipid-lowering drugs were considered hyperlipidemic.^12^ Estimated glomerular filtration rate (GFR) was calculated using the Chronic Kidney Disease Epidemiology Collaboration (CKD-EPI) equation.^13^ Body mass index (BMI) was calculated using the values of body weight (kg) divided by height squared (m) (Quetelet Index). Body surface area (BSA) was calculated as the square root of the product of weight (kg) and height (cm) divided by 3600.^14^ Participants’ alcohol, tobacco, and medication use were recorded.

Routine biochemical tests, lipid profiles, thyroid function tests, and complete blood count data were recorded for all the participants. Rhythm status was classified as either sinus rhythm or atrial fibrillation/flutter. For individuals with sinus rhythm, rhythm-holter monitoring was conducted to detect possible arrhythmic events, and those with atrial fibrillation were recorded.

### Echocardiographic parameters

Transthoracic echocardiographic examination was performed on all patients using a 2.5-3.25 MHz transducer (Philips Affiniti 50 S4-2 Probe system, Andover-USA) following the recommendations of the American Society of Echocardiography.^15-17^ Left ventricular ejection fraction was calculated using the modified Simpson’s method.^18^ Left ventricular mass (g) was calculated using the Devereux formula.^19^ Left ventricular mass index was calculated by dividing left ventricular mass by the body surface area. Left ventricular hypertrophy was defined as a left ventricular mass index of >95 g/m^2^ for women and >115 g/m^2^ for men. Relative wall thickness was calculated as twice the posterior wall thickness divided by the left ventricular diastolic diameter, and the left ventricular geometry was categorized into four categories: normal geometry, concentric remodeling, concentric hypertrophy, and eccentric hypertrophy.^20^

### Chest X-Ray evaluation

Posteroanterior chest radiographs were obtained while the patient was in a standing position. The focal patient distance was 150 cm. Automated exposure control with a fixed tube voltage of 117 kV was used. AAC was graded as follows: grade 0, no visible calcification; grade 1, small spots of calcification or thin calcification on the aortic arch; grade 2, one or more areas of thickened calcification; and grade 3, circular calcification of the aortic arch (Figure 1).^21^

**Figure 1:** – Four-grade score for aortic arch calcification. A) No Calcification, B) Grade I Calcificaton, C) Grade II Calcification, D) Grade III Calcification.

### Stroke parameters

The Trial of Org 10172 in Acute Stroke Treatment (TOAST) classification was used as an etiological framework for ischemic stroke, and patients were evaluated within five subgroups:^22^ 1) large artery atherosclerosis, 2) cardioembolism, 3) small vessel occlusion, 4) stroke of other determined etiology, and 5) stroke of undetermined etiology. Stroke severity was determined using the National Institutes of Health Stroke Score.^23^ Bamford classification^24^ was used to assess the affected vascular territory by dividing it into four classes: total anterior circulation stroke, partial anterior circulation stroke, lacunar syndrome, and posterior circulation syndrome. The Modified Rankin Scale (mRS) was used to assess post-stroke disability and evaluate functional recovery.^25^

### Statistical analysis

The IBM SPSS Statistics software (IBM Corp. Released 2011. IBM SPSS Statistics for Windows, Version 20.0. Armonk, NY: IBM Corp) was used for all statistical analyses. The normal distribution of continuous variables was assessed by visual examination of histograms, Q–Q plots, and the Kolmogorov-Smirnov test. Normally distributed continuous variables were presented as mean (± standard deviation), non-normally distributed continuous variables as median (interquartile range), and categorical variables as numbers and percentages. The Student’s t-test (unpaired) was used to compare normally distributed continuous variables between the two groups, while the Mann-Whitney U test was used for non-normally distributed continuous variables. Categorical variables were compared using chi-square or Fisher’s exact tests. The Kappa value was used to calculate interobserver variability. A logistic regression analysis was performed to compare the relationship between AAC and recurrent cerebrovascular events. In the univariate regression analysis, a two-sided p-value of less than 0.1 was considered statistically significant for inclusion in the multivariate regression analysis. Pearson or Spearman tests were used to analyze the correlations between the parameters. The relationship between AAC and recurrent cerebrovascular events was evaluated using a Receiver Operating Characteristic (ROC) curve analysis. A two-sided p-value < 0.05 was considered statistically significant for all comparisons.

## Results

In total, 409 patients were included in the study during the specified period. The following patients were excluded from the study: previous stroke history (n = 62), transient ischemic attack (n = 53), hemorrhagic stroke (n = 51), malignancy (n = 4), pregnancy (n = 2), active infection (n = 6), and improper chest radiography (n = 7). Eleven patients refused to participate in the study, and 10 were lost to follow-up. The remaining 203 patients were included in the study (Figure 2). Of these, 95 were female, accounting for 46.8% of the total population. The average age of the participants was 69 (±12.3) years. The basic demographic characteristics of the study participants are presented in Table 1.

**Figure 2:** – Flow chart for patient selection. CVD: cardiovascular disease; TIA: transient ischemic attack.

**Table 1:** – Clinical and demographic characteristics of the sample

Characteristic	Total (n=203)	Non-recurrent Stroke (n=154)	Recurrent Stroke (n=49)	p
Gender (Female), n (%)	95 (46.8%)	72 (46.8%)	23 (46.9%)	0.556
Age (year)	69 (±12.3)	68.4 (±12.6)	71.1 (±11.2)	0.231
Weight (kg)	75 (15)	76 (15)	75 (14)	0.964
Height (cm)	165 (12)	166 (12)	165 (16)	0.935
BMI (kg/m2)	27.1 (5.1)	27.0 (5.5)	27.1 (4.0)	0.958
BSA (m2)	1.87 (±0.17)	1.87 (±0.17)	1.86 (±0.17)	0.941
Hypertension, n (%)	156 (77.2%%)	110 (71.4%)	46 (93.9%)	0.001
Diabetes Mellitus, n (%)	75 (36.9%)	53 (34.4%)	22 (44.9%)	0.125
Coronary Artery Disease, n (%)	42 (20.7%)	30 (19.5%)	12 (24.5%)	0.286
Chronic Kidney Disease, n (%)	10 (4.9%)	7 (4.5%)	3 (6.1%)	0.45
Hyperlipidemia, n (%)	97 (47.8%)	73 (47.4%)	24 (49%)	0.488
Cigarette, n (%)	57 (28.1%)	41 (26.6%)	16 (32.7%)	0.26
Alcohol, n (%)	19 (9.4%)	15 (9.7%)	4 (8.2%)	0.497
Renin-Angiotensin System Inhibitor, n (%)	122 (60.1%)	84 (54.5%)	38 (77.6%)	0.003
Statin, n (%)	119 58.6%)	91 (59.1%)	28 (57.1%)	0.468
Calcium Channel Blocker, n (%)	61 (30%)	42 (27.3%)	19 (38.8%)	0.09
Beta-Blocker, n (%)	76 (37.4%)	54 (35.1%)	22 (44.9%)	0.143
Insulin, n (%)	17 (8.4%)	12 (7.8%)	5 (10.2%)	0.392
Anticoagulant, n (%)	34 (16.7%)	20 (13%)	14 (28.6%)	0.012
Fibrate, n (%)	3 (1.4%)	1 (0.6%)	2 (4.1%)	0.145
Antiplatelet, n (%)	78 (38.8%)	58 (37.7%)	20 (40.8%)	0.433
Acetylsalicylic Acid, n (%)	61 (30%)	108 (70.1%)	34 (69.4%)	0.527
Oral Antidiabetic, n (%)	62 (30.5%)	43 (27.9%)	19 (38.8%)	0.105
Atrial Fibrillation, n (%)	42 (20.7%)	34 (22.1%)	15 (30.6%)	0.042
CHA_2_DS_2_-VASc Score	4 (1)	4 (2)	5 (2)	<0.001
**Aortic Arch Calcification**				
0	98 (48.3%)	86 (55.8%)	12 (24.5%)	<0.001
1	71 (35%)	49 (31.8%)	22 (44.9%)	
2	31 (15.3%)	17 (11%)	14 (28.6%)	
3	3 (1.5%)	2 (1.3%)	1 (2.0%)	
≥1	105 (51.7%)	68 (44.1%)	37 (75.5%)	<0.001
≥2	24 (16.7%)	19 (12.3%)	15 (30.6%)	0.004
**Bamford Classification**				
LACS, n(%)	59 (29.1%)	49(31.2%)	10(20.4%)	
PACS, n(%)	83 (40.9%)	55(35.7%)	28(57.1%)	
POCS, n(%)	46 (22.7%)	38(24.7%)	8(16.3%)	0.118
TACS, n(%)	14 (6.9%)	11(7.1%)	3(6.1%)	
**TOAST Classification**				
Large Artery Atherosclerosis, n (%)	54 (26.6%)	37 (24.0%)	17 (34.7%)	
Lacunar, n (%)	42 (20.7%)	28 (18.2%)	14 (28.6%)	
Cardioembolism, n (%)	20 (9.9%)	17 (11.0%)	3 (6.1%)	
Other, n (%)	5 (2.5%)	3 (1.9%)	2 (4.0%)	0.138
Undetermined, n (%)	81 (39.9%)	68 (44.1%)	13 (26.5%)	
Modified Rankin Scale	2 (3)	1 (3)	2 (2)	0.021
NIH Stroke Scale	3 (5)	3 (4)	5 (7)	0.047

*BMI: body mass index; BSA: body surface area.*

In this study, 156 individuals (77.2%) had hypertension, and this proportion was significantly higher in the recurrent cerebrovascular event group (93.9%, 46 individuals) than in the non-recurrent group (110 individuals, 71.4%) (p<0.001). Moreover, the use of renin-angiotensin system blockers (77.6%) and anticoagulants (28.6%) was significantly higher in the recurrent event group than in the other groups (p=0.003 and p=0.012, respectively). Atrial fibrillation was detected in 34 individuals (22.1%) in the first cerebrovascular event group and 15 individuals (30.6%) in the recurrent event group, showing a significant difference (p=0.042). Additionally, the calculated CHA_2_DS_2_-VASc score, irrespective of rhythm, was higher in the recurrent cerebrovascular events group, with a median of 5 (2), than in the other groups (p<0.001).

At the end of the first year of follow-up, recurrent stroke was observed in 49 patients (24.1%) patients. The patients were divided into two groups based on their stroke recurrence status (non-recurrent stroke, 154 patients; recurrent stroke, 49 patients). The two groups had similar demographic characteristics. A comparison of the two groups is presented in Table 1. Regarding neurological characteristics, partial anterior circulation stroke was the most common subtype of stroke. It was found in 55 individuals (35.7%) in the non-recurrent stroke group and 28 individuals (57.1%) in the recurrent event group. The groups were similar according to the Bamford classification. In terms of the TOAST classification, cases with an unknown cause predominated the entire group, which was notable. This pattern was observed not only in individuals experiencing their first cerebrovascular event but also in 17 individuals (34.7%) with large artery atherosclerosis and 14 individuals (28.6%) with lacunar infarcts among those with recurrent cerebrovascular events. Stroke cases with an undetermined etiology were ranked third. However, these differences were not statistically significant. The modified Rankin and NIH Stroke Scale scores were higher in the recurrent cerebrovascular event group (p=0.021 and 0.047, respectively).

AAC was compared between the groups. AAC was detected in 105 (51.7%) patients. There was a significant difference in the AAC grades between the groups (p<0.001). One patient (2%) had grade 3 AAC and 14 (28.6%) patients had grade 2 AAC in the recurrent CVA group. These numbers were only 2 (1.3%) with grade 3 AAC and 17 (11%) with grade 2 AAC in the nonrecurrent cerebrovascular event group. Therefore, patients with AAC were combined and analyzed as a single group. AAC (grade ≥1) was observed in 37 individuals (75.5%) in the AAC cerebrovascular event group and 68 (44.1%) in the non-recurrent cerebrovascular event group. The recurrent cerebrovascular event group had a significantly higher AAC prevalence (p<0.001). Among the participants, 60 randomly selected chest radiographs were evaluated by two cardiologists and one neurologist, who were blinded to the study findings for interobserver variability, and there was a reasonably high degree of consistency between assessments. (Kappa value = 0.816, p < 0.001).

Laboratory and echocardiographic features of each group are shown in Table 2. In the recurrent cerebrovascular events group, the glomerular filtration rate (GFR) was statistically significantly lower compared to the non-recurrent cerebrovascular events group [77 mL/min/1.73 m^2^ (33) vs. 85 mL/min/1.73 m^2^ (23), p=0.018]. Additionally, the red cell distribution width (RDW) was significantly higher in the recurrent cerebrovascular event group (16.3 [3.8] fL) than in the non-recurrent cerebrovascular event group (15.2 [2.1] fL) (p=0.001). No statistically significant differences were observed between the groups in terms of other laboratory parameters.

**Table 2:** – Laboratory and Echocardiographic findings of the study population

Parameters	Non-recurrent Stroke	Recurrent Stroke	p
**Laboratory parameters**			
Glucose (mg/dL)	111 (50)	117 (40)	0.541
GFR (mL/min/1.73 m^2^)	85 (23)	77 (33)	0.018
Alanine Aminotransferase (U/L)	15 (11)	15 (18)	0.737
Aspartate Aminotransferase (U/L)	18 (8)	18 (13)	0.372
Sodium (mEq/L)	140 (3)	140 (5)	0.657
Potassium (mEq/L)	4.3 (±0.5)	4.3 (±0.5)	0.768
Triglyceride (mg/dL)	135 (98)	128 (74)	0.916
Total cholesterol (mg/dL)	186 (±44.4)	178 (±51)	0.185
Low-density lipoprotein (mg/dL)	111 (±37.3)	108 (±41.4)	0.561
High-density lipoprotein (mg/dL)	42 (18)	42 (16)	0.632
White Blood Cell (n, x10^3)^	8.15 (±2.44)	8.20 (±2.87)	0.924
Hemoglobin (g/dL)	13.0 (±2)	12.5 (±2.3)	0.112
Platelet (n, x103)	233 (96)	249 (84)	0.052
PDW (%)	16.7 (±2.6)	16.5 (±2.5)	0.509
Plateletcrit (%)	0.22 (0.07)	0.24 (0.09)	0.047
Neutrophil (n, x10^3)^	4.75 (2.22)	4.60 (2.88)	0.881
Lymphocyte (n, x10^3)^	2.05 (±0.69)	1.95 (±0.86)	0.141
RDW (fL)	15.2 (2.1)	16.3 (3.8)	0.001
Free T3 (ng/L)	2.45 (0.64)	2.37 (0.80)	0.261
Free T4 (ng/dL)	1.02 (0.20)	1.03 (0.22)	0.528
TSH (mIU/L)	1.36 (1.32)	1.33 (1.28)	0.885
**Echocardiographic parameters**			
Left Ventricular End-Diastolic Diameter (mm)	44 (4)	44 (4)	0.4
Left Ventricular End-Systolic Diameter (mm)	29 (4)	29 (4)	0.691
Left Ventricular Ejection Fraction (%)	59.88 (6.3)	59.6 (8.2)	0.75
Left Ventricular Mass (gr)	203.05 (69)	207.28 (67)	0.949
Left Ventricular Mass Index (gr/m^2^)	109.49 (31.7)	109 (38.1)	0.991
Relative Wall Thickness	0.54 (±0.07)	0.55 (±0.07)	0.574
Aortic Diameter (mm)	33.46 (±3.9)	33.49 (±4.3)	0.908
Interventricular Septal Thickness (mm)	12 (2)	13 (3)	0.36
Posterior Wall Thickness (mm)	12 (2)	12 (2)	0.563
Left Atrial Diameter (mm)	36 (5)	37 (4)	0.275
Estimated Pulmonary Artery Pressure (mmHg)	9 (15.8)	16 (17.8)	0.009
Aortic Regurgitation (≥moderate), n (%)	2 (1.3)	3 (6.1)	0.058
Mitral Regurgitation (≥moderate), n (%)	4 (2.6)	3 (6.1)	0.363
Tricuspid Regurgitation (≥moderate), n (%)	6 (3.9)	2 (4.1)	0.616
**Left Ventricular Geometry**			
Concentric Hypertrophy	82 (53.2)	26 (53.1)	
Concentric Remodeling	64 (41.6)	21 (42.9)	0.991
Eccentric Hypertrophy	4 (2.6)	1 (2)	
Normal Geometry	4 (2.6)	1 (2)	

*GFR: Glomerular Filtration Rate; RDW: Red Blood Cell Distribution Width; PDW: Platelet distribution width; TSH: Thyroid Stimulating Hormone.*

The groups were similar in terms of echocardiographic parameters, except for estimated pulmonary artery pressure. The estimated pulmonary artery pressure was higher in the recurrent stroke group [16 (±17.8) mmHg] than in the non-recurrent stroke group [9 (±15.8) mmHg]), and this difference was statistically significant (p=0.009).

AAC, RDW, and aortic regurgitation were associated with recurrent stroke in univariate logistic regression analysis (p<0.1) (Table 3). In the multivariate regression analysis, an independent and strong association was found between AAC (OR 3.544, p<0.001), RDW (OR 1.214, p=0.008), and stroke (Table 3, Central Illustration).

**Table 3:** – Univariate and Multivariate analysis for non-dipper blood pressure pattern

Parameter	Univariate analysis		Multivariate analysis		
	β	p	β	p	Hazard Ratio (95% CI)
Hypertension	1.239	0.130			
Atrial Fibrillation	-0.286	0.751			
Aortic Arch Calcification (≥1)	1.260	0.007	1.265	0.001	3.544 (1.653 - 7.598)
Glomerular Filtration Rate	-0.008	0.432			
Estimated Pulmonary Artery Pressure	0.015	0.190			
NIH Stroke Scale	-0.041	0.567			
Modified Rankin Scale	0.201	0.398			
Anticoagulant	0.863	0.174			
RAS Inhibitor	0.259	0.621			
CHA_2_DS_2_-VASc Score	-0.128	0.564			
RDW	0.135	0.070	0.194	0.008	1.214 (1.053- 1.400)
Aortic Regurgitation	2.270	0.097	2.376	0.066	10.766 (0.857 - 135.171)
Constant	-4.652	0.004			

*CI: Confidence Interval*

Correlation analysis was performed to further investigate the potential association between AAC and RDW. Patients with AAC had a higher RDW (p=0.014). There was no correlation between the AAC grade and RDW (p=0.055, r=0.135). However, there was a moderate correlation between the AAC grade and recurrent stroke (p<0.001, r=0.277). These results suggest that RDW is not a significant confounder.

ROC curve analysis yielded a strong predictive ability of AAC grade ≥ 1 for recurrent stroke (AUC=0.657, p<0.001) (Figure 3). The presence of AAC on chest radiography had sensitivity and specificity of 75.5% and 55.8%, respectively, for recurrent stroke (Table 4).

**Figure 3:** – Receiver operating characteristic curve of aortic arch calcification for recurrent stroke.

**Table 4:** – Area Under the Curve for Aortic Arch Calcification

AUC	p	95% CI	Sensitivity	Specificity	PPV	NPV
0.657	0.001	0.572 - 0.742	0.755	0.558	33.33%	87.76%

*AUC: area Under the curve; CI: confidence interval; PPV: positive predictive value; NPV: negative predictive value.*

## Discussion

This study aimed to evaluate potential risk factors for recurrent stroke and determine their association with AAC. Consistent with these data, aortic calcification, which is a specific subgroup of this condition, was found to be a predictor of both incident and recurrent strokes, which is a specific subgroup of this condition.^7^ Additionally, it has been demonstrated that the RDW value is higher in patients with recurrent stroke. These findings may serve as clinical guidelines.

This study included patients with any form of stroke. Therefore, it is expected that there will be no significant difference in classical risk factors. However, an interesting finding is worth noting in the results. In the recurrent stroke group, hypertension was found to be significantly more prevalent at a statistically significant level. Hypertension was the most common cardiovascular risk factor. Although it is generally considered the most important risk factor for the first stroke, its role in the risk of recurrence remains unclear. Although it is an independent risk factor for cerebrovascular events, it also poses a risk within the spectrum of cerebrovascular events. If subgrouping and classification were performed for other risk factors, differences could have been detected. However, it is important to bear in mind that hypertension remains a risk factor for strokes. We believe that the observed difference in the use of RAS blockers between groups also arises from this clinical entity.

The higher incidence of atrial fibrillation and greater use of anticoagulant drugs in the recurrent stroke group were considered to be a result of the relationship between these clinical conditions. The CHA_2_DS_2_-VASc score was evaluated, regardless of the presence of AF in the groups. A significantly higher median CHA_2_DS_2_–VASc score was observed in the recurrent stroke group. As shown in Table 1, in addition to hypertension, the groups were similar, and we believe that this difference can be attributed to hypertension, which is a component of the CHA_2_DS_2_-VASc score calculation.

In the recurrent stroke group, a lower GFR was observed. This could be attributed to the fact that the individuals in the recurrent stroke group had poorer performance and higher morbidity, which may have led to a higher prevalence of nutritional issues. Consequently, loss of muscle mass can affect serum creatinine levels and the GFR.

In a large meta-analysis that included ten studies, predictors of recurrent stroke were examined, and a history of stroke or transient ischemic attack (TIA) and the presence of significant large artery atherosclerosis were found to be associated with recurrent stroke. In this meta-analysis, the findings were assessed based on MRI findings, and the absence of a study that evaluated them using CT or USG has also been discussed.^26^ In addition to this meta-analysis, a recent study investigated the relationship between aortic calcification on thoracic computed tomography and cerebrovascular events. In this study, AAC grade ≥ 1 was associated with recurrent stroke.^27^ This study also mentioned studies conducted using X-rays; however, these studies did not classify AAC in the same manner as in the current study. Additionally, specialized conditions such as the Agatston score were not included in this study. While thoracic computed tomography is certainly superior to chest radiography in a cross-sectional manner, considering the findings obtained from our study, it can be argued that chest radiography is more cost-effective. Further research could explore the concordance between studies that evaluated the two modalities. Indeed, readily available chest radiographs can be used to determine a patient’s risk of recurrent stroke. Chest X-rays are often a part of routine hospital admissions in many clinical settings.

If we consider the concept of “large artery atherosclerosis” mentioned in the meta-analysis, the aorta is the starting point of these arteries. AAC is an important indicator of large artery atherosclerosis. The hypothesis of our study is consistent with the findings of this meta-analysis. In our study, an undetermined etiology was most common in the non-recurrent stroke group (44.1%), whereas large artery atherosclerosis was observed at a higher rate in the recurrent stroke group (34.7%). This difference was not statistically significant, possibly due to the small number of potential participants (p=0.138). Another important point is that chest X-rays are more cost-effective, involve less radiation exposure, and are more readily applicable than tomographic and angiographic modalities. Furthermore, in light of the pandemic, chest X-rays are routinely performed in many healthcare centers during hospitalization as a preferred clinical practice.

Hypertension has a significant effect on the vascular bed. First, increased vascular pressure leads to vascular calcification, an important indicator of hypertension-related vascular damage. The occlusive effect of hypertension plays a role in the etiology of ischemic stroke. Additionally, pressure load can cause rupture and hemorrhage of cerebral vessels, as well as impaired blood supply to the brain tissue owing to compression effects.^28^ Other factors that play a role in occlusive vascular disease through vascular calcification include inflammation, oxidative stress, advanced age, and the renin-angiotensin system.^29-31^ Vascular smooth muscle cells, similar to osteoblasts, are derived from mesenchymal cells. Under the influence of these factors, muscle cells undergo a phenotypic transition to an osteoblast-like phenotype and produce calcium. Thus, vascular calcification begins with the production of calcium in the intima or media of the blood vessel walls.^32^ AAC leads to increased arterial stiffness, resulting in decreased vascular compliance, and is associated with left ventricular hypertrophy and diastolic dysfunction.^33,34^ As a result, cerebral blood flow decreases, leading to impaired nutrition in the related brain regions. For example, a strong correlation has been reported between AAC and renal artery calcification, a significant indicator of renal artery disease. There are two important findings regarding the relationship between recurrent stroke and AAC. AAC is a significant prototype of vascular calcification, which is a systemic condition that affects the entire vascular bed. Therefore, it is an indicator of the risk in patients with ischemic stroke. This indicated that the calcification process was progressive. In fact, these patients experienced recurrent strokes. This finding is important in demonstrating end-organ damage, which is a crucial endpoint in cardiovascular diseases, as it has resulted in clinical outcomes, such as recurrent stroke.

Red cell distribution width (RDW) reflects the heterogeneity of red blood cell volume. In our study, higher RDW values were found in the recurrent stroke group, and this difference was statistically significant in predicting recurrent strokes. The relationship between RDW and the risk of ischemic stroke, carotid artery disease, and cerebral emboli has been reported previously.^35,36^ Additionally, it has been shown that every 1% increase in RDW is associated with a 13% increase in the risk of ischemic stroke.^37^ Our data, consistent with those of other studies, provide additional insights. Our findings are consistent with those of a recent study by Shen et al., which used 5-year follow-up data. Similar to our study, Shen et al. demonstrated a positive association between high RDW levels and an increased risk of recurrent ischemic stroke.^38^

The exact pathophysiological relationship between RDW and recurrent stroke has not been studied extensively. However, similar to AAC, it has been suggested that inflammation and oxidative stress may play a role in the mechanism. Consequently, the survival rate of red blood cells decreases, and erythropoietin production is inhibited, resulting in increased RDW levels.^39^

This study had certain limitations. First, it was conducted at a single center with a relatively small sample size considering cardiovascular diseases. These findings were based on observational data and could not be controlled for confounding variables. In addition, the causal relationship between AAC and recurrent stroke must be determined according to pathophysiological mechanisms. AAC was evaluated for its presence; however, other characteristics such as overall thickness or high-risk features such as ulceration were not evaluated. Furthermore, the overall NIHSS scores were low to moderate, which may limit the generalizability of the results to patients with more severe strokes. Finally, patients with a history of stroke and TIA were excluded, and these data may not apply to this patient population.

## Conclusion

In this study, a statistically significant relationship was observed between recurrent strokes and AAC during one-year follow-up despite the small number of patients. In addition, a relationship was observed between recurrent stroke and RDW. Both parameters are easily accessible and clinically convenient and can provide benefits to clinicians in the monitoring of patients with stroke.

### Highlights

- Recurrent stroke is an important cause of morbidity and mortality.
- AAC is an important clinical marker of vascular disease burden.
- AAC on chest radiography can be a key factor in recurrent strokes.
- RDW has been shown to be an effective indicator of recurrent stroke.

### Ethics

This study complied with the internationally accepted standards for research practice and reporting. This study was approved by the Tekirdag City Hospital Non-Interventional Clinical Research Ethics Committee (ID #26). Written informed consent was obtained from all the participants following the ethical principles of human research outlined in the Declaration of Helsinki.
